# A molecular and epidemiological study of *Vibrio cholerae* isolates from cholera outbreaks in southern Ghana

**DOI:** 10.1371/journal.pone.0236016

**Published:** 2020-07-10

**Authors:** Emelia Konadu Danso, Prince Asare, Isaac Darko Otchere, Lorenzo Moses Akyeh, Adwoa Asante-Poku, Samuel Yaw Aboagye, Stephen Osei-Wusu, David Opare, Francine Ntoumi, Alimuddin Zumla, Samuel Duodu, Dorothy Yeboah-Manu

**Affiliations:** 1 Noguchi Memorial Institute for Medical Research, College of Health Sciences, University of Ghana, Legon, Accra, Ghana; 2 West African Centre for Cell Biology of Infectious Pathogens, University of Ghana Legon, Legon, Accra, Ghana; 3 Department of Biochemistry, Cell and Molecular Biology, University of Ghana, Legon, Accra, Ghana; 4 Institute for Environment and Sanitation Studies, University of Ghana, Legon, Accra, Ghana; 5 National Public Health and Reference Laboratory, Accra, Ghana; 6 Université Marien NGouabi, Fondation Congolaise pour la Recherche Médicale (FCRM), Brazzaville, Congo; 7 Institute for Tropical Medicine, University of Tübingen, Tübingen, Germany; 8 Division of Infection and Immunity, University College London and NIHR Biomedical Research Centre, UCL Hospitals NHS Foundation Trust, London, England, United Kingdom; Nitte University, INDIA

## Abstract

Cholera remains a major global public health threat and continuous emergence of new *Vibrio cholerae* strains is of major concern. We conducted a molecular epidemiological study to detect virulence markers and antimicrobial resistance patterns of *V*. *cholerae* isolates obtained from the 2012–2015 cholera outbreaks in Ghana. Archived clinical isolates obtained from the 2012, 2014 and 2015 cholera outbreaks in Ghana were revived by culture and subjected to microscopy, biochemical identification, serotyping, antibiotic susceptibility testing, molecular detection of distinct virulence factors and Multi-Locus Variable-Number of Tandem-Repeat Analysis (MLVA). Of 277 isolates analysed, 168 (60.6%) were confirmed to be *V*. *cholerae* and 109 (39.4%) isolates constituted other bacteria (*Escherichia coli*, *Aeromonas sobria*, *Pseudomonas aeruginosa*, *Enterobacter cloacae* and *Enterococci faecalis*). Serotyping the *V*. *cholerae* isolates identified 151 (89.9%) as Ogawa, 3 (1.8%) as Inaba and 14 (8.3%) as non-O1/O139 serogroup. The O1 serogroup isolates (154/168, 91.7%) carried the cholera toxin *ctxB* gene as detected by PCR. Additional virulence genes detected include *zot*, *tcpA*, *ace*, *rtxC*, *toxR*, *rtxA*, *tcpP*, *hlyA* and *tagA*. The most common and rare virulence factors detected among the isolates were *rtxC* (165 isolates) and *tcpP* (50 isolates) respectively. All isolates from 2014 and 2015 were multidrug resistant against the selected antibiotics. MLVA differentiated the isolates into 2 large unique clones A and B, with each predominating in a particular year. Spatial analysis showed clustering of most isolates at Ablekuma sub-district. Identification of several virulence genes among the two different genotypes of *V*. *cholerae* isolates and resistance to first- and second-line antibiotics, calls for scaleup of preventive strategies to reduce transmission, and strengthening of public health laboratories for rapid antimicrobial susceptibility testing to guide accurate treatment. Our findings support the current WHO licensed cholera vaccines which include both O1 Inaba and Ogawa serotypes.

## 1. Introduction

Cholera is a highly virulent, lethal diarrhoeal disease caused by ingestion of water or food contaminated with the bacterium, *Vibrio cholerae (V*. *cholerae)*. Vibrios can survive for a long time in coastal waters contaminated by human faeces. Seven cholera pandemics have been experienced globally and it continues to cause outbreaks locally and regionally across the African continent [1, 2]. The World Health Organization (WHO) estimates that in 2018, there were 1.3 to 4.0 million cases and 21,000 to 143,000 deaths due to cholera globally [1]. The requirements for preventing outbreaks include improvement in sanitation, provision of safe drinking water, and safe food and water practices as well as hand washing. These are difficult to achieve in developing countries including Ghana, where annual cycles of cholera outbreaks continue to occur. Since the first reported outbreak in 1970, Ghana has experienced many epidemics [2, 3]. Over the last two decades, the country reported an average of 3,066 (range: 50–10,628) cholera cases with case fatality rate of 1.7%, though the WHO estimates more than 40,000 cases occur annually in years of large outbreaks [3]. The 2014 outbreak, which recorded over 28,900 cases and 243 deaths in Ghana was the worst ever affecting all the 10 regions with 72% of cases occurring in the Greater Accra region [4]. Later in 2016, a small wave of the disease was experienced in the central region of Ghana accounting for 596 cases [5]. While oral rehydration therapy is critical for case management, complementing with antibiotics to eradicate vibrios is important in the management, prevention of spread and control of the disease [6]. Recent reports indicate the emergence of antibiotic resistant *V*. *cholerae* strains from cholera-endemic African countries [7, 8].

The current licenced cholera vaccine contains limited variants of killed whole cells of the O1 *V*. *cholerae* serogroup, at the same time newer variants are emerging with non-O1 types causing disease in some geographical areas [9]. Moreover, the presence of distinct genetic virulence markers influences clinical cholera. This implies that the inter serotype polymorphism in virulence markers may affect the efficacy of vaccines with limited strain composition in specific geographical areas. Therefore, a molecular epidemiological study to understand the occurrence of distinct markers among outbreak isolates is important for future vaccine development. Moreover, utilization of discriminating tools such as multi-locus variable number of tandem repeats analysis (MLVA) will aid in tracking disease spread, which is crucial for disease control. Furthermore, the emergence of antimicrobial drug resistance, makes it judicious to routinely monitor changes in strain susceptibility to antimicrobial drugs hence adapt to treatment recommendations. We conducted a molecular epidemiological study to detect virulence markers and antimicrobial resistance patterns of *V*. *cholerae* isolates obtained from cholera cases occurring during the 2012–2015 outbreaks in Ghana.

## 2. Materials and methods

### 2.1. Isolate revival and characterisation

Archived clinical isolates used in this study were obtained from presumptive cholera cases attending some selected health facilities (Ga West Municipal hospital, Achimota hospital, Korle-Bu Polyclinic, La General hospital, University of Ghana hospital, Princess Marie Luis children’s hospital, and 37 Military hospital) in Southern Ghana during the 2012, 2014 and 2015 cholera outbreak years.

The isolates were concurrently sub-cultured on thiosulphate-citrate-bile salt-sucrose (TCBS) agar, blood agar and in alkaline peptone water and all three media were aerobically incubated overnight at 37° C. Colonies were subjected to Analytical profile index (API 20E) standard biochemical tests. O1 polyvalent anti-sera and monovalent anti-sera were used to define isolates as Ogawa, Inaba or Hikojima serotype. Isolates that were non-O1 were further tested using O139 anti-sera. *Vibrio cholerae* isolates were genetically confirmed by the presence of *ompW* and/or *ctxB* genes using PCR.

### 2.2. Drug susceptibility testing

Phenotypic drug susceptibility testing by Kirby-Bauer disc diffusion method was performed using Muller-Hinton Agar in accordance with the Clinical and Laboratory Standards Institute (CLSI) guidelines [10]. The antibiotic susceptibility profiles of the isolates were determined against Cotrimoxazole (23.75μg/1.25μg), Ampicillin (10μg), Tetracycline (30μg), Ciprofloxacin (5μg), Amikacin (30μg), Gentamicin (10μg), Erythromycin (15μg), Streptomycin (10μg), Cefuroxime (30μg), Ceftriaxone (30μg), Nalidixic acid (30μg), Doxycyclin (30μg), Azetromycin (30μg), Flucloxacillin (5μg), cefotaxime (30μg) and ceftazidime (30μg) and Chloramphenicol (30μg). The reference strain *Escherichia coli* ATCC 25922 was used as a control.

### 2.3. Extended-spectrum β-lactamases (ESBLs)

Each bacterial isolate was first screened for Extended Spectrum Beta Lactamase (ESBL) production by the disc diffusion method using the antibiotics ceftriaxone (30μg), cefotaxime (30 μg), ceftazidime (30 μg) and aztreonam according to CLSI guidelines [10]. An isolate with reduced susceptibility to any of the four antibiotics was confirmed following the recommended CLSI double disc confirmatory method [10], the modified CLSI confirmatory test [11] and a combination disc method using discs of cefepime alone and in combination with clavulanic acid [12]. All screened-positive isolates were first tested with the CLSI confirmatory test using discs of ceftazidime and ceftaxime alone and in combination with clavulanic acid. *Escherichia coli* ATCC 25922 and *Klebsiella pneumonia* ATCC 700603 were used as negative and positive controls respectively.

### 2.4. DNA extraction

*V*. *cholerae* cell lysate was used as DNA template as previously described by Nandi *et al*. [13]. Briefly, two loops full (10 μL) of biochemically confirmed colonies of *V*. *cholerae* from a 24-hour nutrient agar culture was suspended in 100 μL of nuclease free water. The bacterial suspension was heated at 95°C for 10 minutes to release crude DNA. The DNA was then stored at -20°C for further analysis.

### 2.5. Detection of *V*. *cholerae* virulence genes

Genotypic detection of the following *V*. *cholerae* genes: *ompW*, *ctxB*, *O1-rfb*, *zot*, *toxR*, *tcpA*, *tcpP*, *rxtA*, *rxtC*, *ace*, *hlyA* and *tagA* [14] was carried out using conventional PCR with specific primers (Table 1). Each reaction volume in a total of 25μl comprised of 13.5μl of nuclease free water, 2.5μl (10pmol/μl) of forward primer, 2.5μl (10pmol/μl) of reverse primer, 0.7μl (2.5mM) of dNTPs, 0.3μl (5U/ul) of hot start taq plus polymerase, 2.5μl of 10X PCR coral load buffer (Qiagene) and 3μl of template. The reaction mixture was subjected to 35 cycles of amplification with PCR condition set at; initial denaturation at 95°C for 5min, denaturation at 94°C for 30sec, annealing at specified temperature (Table 1) for 50sec, extension at 72°C for 50sec and final extension after the 35 cycles at 72°C for 5min.

**Table 1:** A list of primers used in this study.

Primer no.	Target gene primer	Primer Sequence	Annealing Temperature Used for PCR	Reference
1	*ompW* forward	CACCAAGAAGGTGACTTTATTGTG	59 °C	[13]
	*ompW* reverse	GAACTTATAACCACCCGCG	59 °C	[13]
2	*O1*-*rfb* forward	GTTTCACTGAACAGATGGG	54 °C	[15]
	*O1*-*rfb* reverse	GGTCATCTGTAAGTACAAC	54 °C	[15]
3	*ctxB* forward	GGTTGCTTCTCATCATCGAACCAC	60 °C	[16]
	*ctxB* reverse	GATACACATAATAGAATTAAGGAT	60 °C	[16]
4	*zot* forward	GCTATCGATATGCTGTCTCCTCAA	62 °C	[17]
	*zot* reverse	AAAGCCGACCAATACAAAAACCAA	62 °C	[17]
5	*toxR* forward	ATGTTCGGATTAGGACAC	54 °C	[18]
	*toxR* reverse	TACTCACACACTTTGATGGC	54 °C	[18]
6	*ace* forward	AGAGCGCTGCATTTATCCTTATTG	64 °C	[19]
	*ace* reverse	AACTCGGTCTCGGCCTCTCGTATC	64 °C	[19]
7	*rtxA* forward	CTG AAT ATG AGT GGG TGA CTT ACG	64 °C	[20]
	*rtxA* reverse	GTG TAT TGT TCG ATA TCC GCT ACG	64 °C	[20]
8	*rtxC* forward	CGA CGA AGA TCA TTG ACG AC	58 °C	[16]
	*rtxC* reverse	CAT CGT CGT TAT GTG GTT GC	58 °C	[16]
9	*tagA* forward	GGTGGTAAGATATTCACTC	57 °C	[20]
	*tagA* reverse	GAGACATCTATAGAATACTGGCTG	57 °C	[20]
10	*tcpA* forward	CACGATAAGAAAACCGGTCAAGAG	64 °C	[14]
	*tcpA* reverse	ACCAAATGCACGCCGAATGGAGC	64 °C	[14]
11	*tcpP* forward	ACTCTGTGAATATCATCCTGCC	59 °C	[21]
	*tcpP* reverse	CTGGGTAAGCCAAACATTGG	59 °C	[21]
12	*hlyA*-Forward	GGC AAA CAG CGA AAC AAA TAC C	62 °C	[22]
	*hlyA*-Reverse	CTC AGC GGG CTA ATA CGG TTT A	62 °C	[22]

### 2.6. Multi-locus variable number of tandem repeat analysis (MLVA)

The MLVA was achieved using five previously described loci by Danin Poleg *et al*. [23]. Five loci including VC0147, VC0436-7, VC1650, VC0171 and VC0283 were amplified using the primers and conditions described by Morita *et al*. [24]. The PCR products were purified and sequenced by outsourcing. We cleaned and analysed the generated sequences using the Staden software package [25]. The numbers of repeats of each VNTR per isolate were digitized and recorded into Microsoft excel and uploaded into the MIRU-VNTR*plus* web application (https://www.miru-vntrplus.org/MIRU/index.faces) for clustering analysis implemented with a neighbour-joining clustering algorithm. The resulting dendrogram was annotated with patient demographic information using the ggtree package implemented in R [26].

### 2.7. Spatial analysis

Spatial analysis was performed using both the MLVA and epidemiological (residential address) data. The GIS co-ordinates of the residential location of the cases were used to construct a graphical plot of the distribution of the *V*. *cholerae* isolates using ArcMap employed in ArcGIS v10.1 [27].

### 2.8. Ethical consideration

Cholera outbreaks are of great public health concern in Ghana; there are control and routine surveillance measures to reduce the epidemics. To understand the transmission dynamics of *V*. *cholerae* and strategize for subsequent spread of the disease in the sub-region, the National Public Health and Reference Laboratory (NPHRL) of the Ghana Health Service, requested the Noguchi Memorial Institute for Medical Research to undertake this study.

## 3. Results

### 3.1. Demographic and clinical characteristics of cases

A total of 277 archived isolates were used of which 43.3% (120) were received from the National Public Health and Reference laboratory, Korle-Bu and the remaining 157 (56.7%) were isolated from human stool samples at the Bacteriology Department of Noguchi Memorial Institute for Medical Research (NMIMR). The isolates were obtained from 152 (54.9%) males and 119 (43.0%) females and 6 (2.1%) with gender not specified. Among the 277 cases, age ranged between 1 month to 84 years; 47 (17.0%), 58 (21.0%) and 170 (61.3%) representing the paediatric (0-5years), older children (6–17 years) and adult (>17years) age respectively.

**Fig 1:**
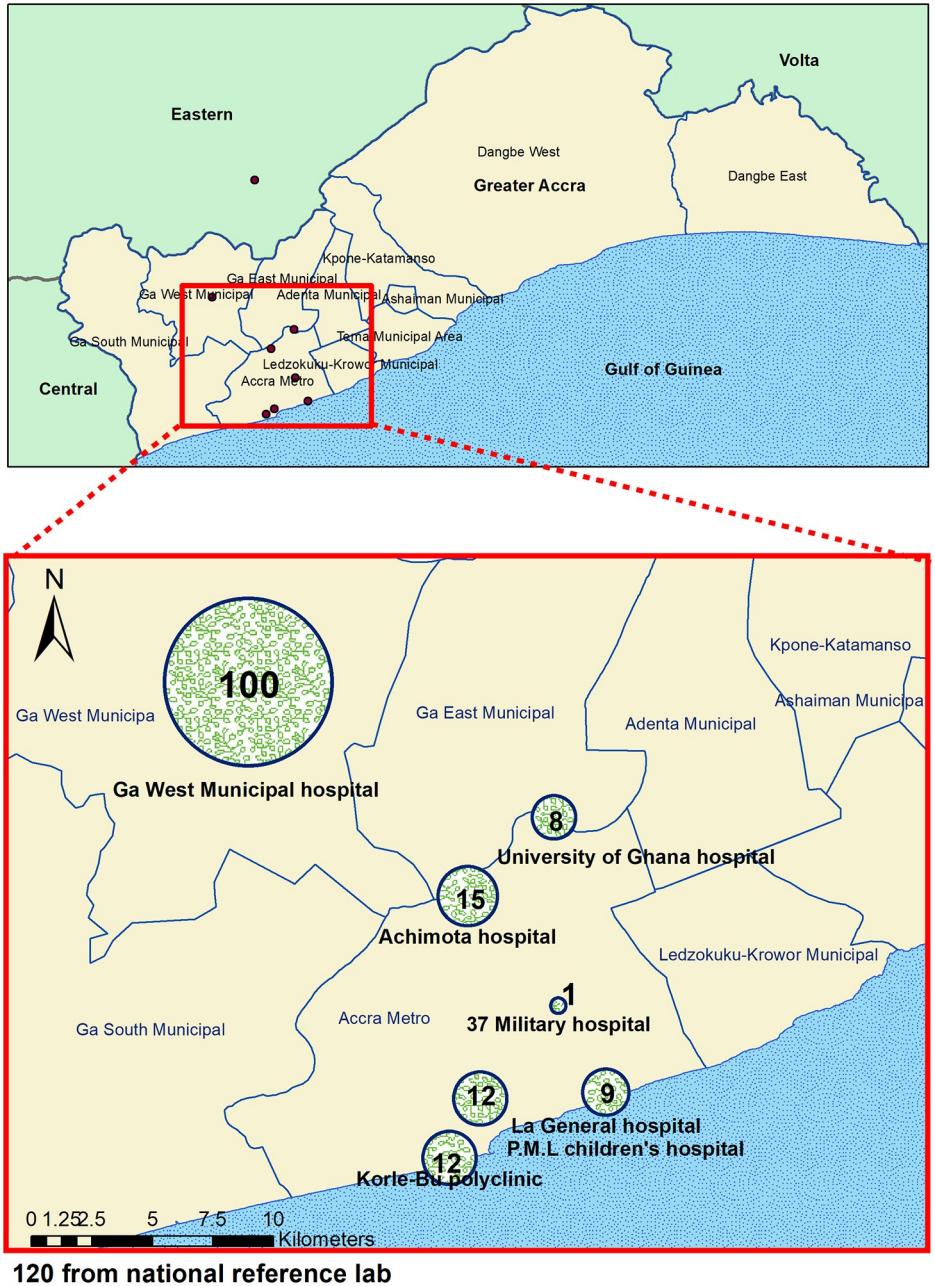
A map showing geographical distribution and the number of isolates of *V*. *cholerae*.

While the isolates from the NPHRL (120) were collected across Ghana, the 157 NMIMR isolates were from selected health facilities in the Greater Accra region specifically, Ga West Municipal hospital 100 (63.7%), Achimota hospital 15 (9.6%), Korle-Bu Polyclinic 12 (7.6%), La General Hospital 9 (5.7%), University of Ghana hospital 8 (5.2%), Princess Marie Luis children’s hospital 12 (7.6%), and 37 Military hospital 1(0.6%) (Fig 1). Clinical records on cases of the NMIMR isolates showed that 71 (45.2%) had taken antibiotics, 13 (8.3%) antibiotics and antimalarial, 17 (10.8%) could not recall medication taken and 56 (35.7%) had not taken any medication prior to seeking medical care. Clinicians’ provisional diagnosis indicated cholera 139 (88.5%), gastroenteritis 6 (3.8%), gastroenteritis/malaria 3 (1.9%), diarrhoea 8 (5.2%) and retroviral infection 1 (0.6%).

### 3.2. Identified species and antimicrobial susceptibility pattern

**Fig 2:**
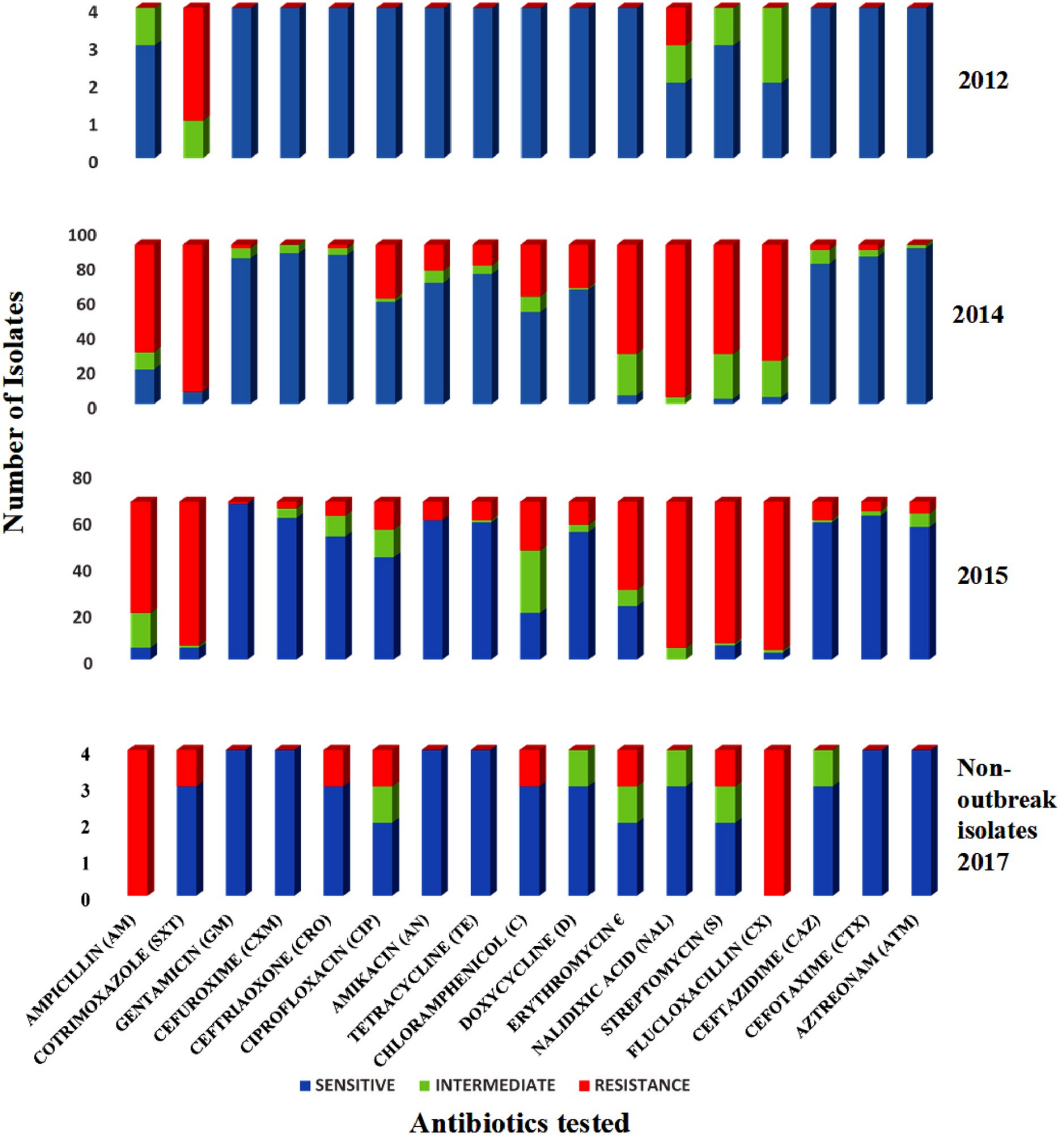
Antimicrobial susceptibility profile of *V*. *cholerae* outbreak and non-outbreak isolates by year of disease onset.

Of the 277 bacteria revived, 168 (61%) were both phenotypically and genotypically characterised as *V*. *cholerae* and the remaining 109 (39%) isolates were identified as: *Aeromonas sobria*: 14 (12.8%), *Vibrio parahaemolyticus*: 2 (1.8%), *Pseudomonas aeruginosa*: 7 (6.4%), *Enterobacter cloacae*: 16 (14.8%), *Enterococci faecalis*: 31 (28.4%), *Escherichia coli*: 26 (23.9%), *Klebsiella pneumonia*: 6 (5.5%) and *Proteus mirabilis*: 7 (6.4%). Since the focus of the study was on *V*. *cholerae*, the other bacteria were not further studied. From the antibiotic susceptibility screening, we observed increasing antibiotic resistance among the *V*. *cholerae* isolates from 2012, 2014 and 2015 cholera outbreak seasons (Fig 2). The 2012 *V*. *cholerae* isolates showed high resistance patterns to sulfamethoxazole-trimethoprim (3/4, 75%) and nalidixic acid (1/4, 25%) but were susceptible to over 70% of the antibiotics (S1 Table). Among the 2014 isolates Azithromycin and Cotrimoxazole were the most and least effective antibiotics respectively. In 2015, Gentamicin and flucloxacilin were respectively the most and least potent antibiotics.

### 3.3. Virulence genes distribution among *V*. *cholerae* isolates

Genotypic assays confirmed all *V*. *cholerae* isolates by the presence of *ompW*. Of the 168: 151 (89.8%), 3 (1.8%) and 14 (8.3%) were of the Ogawa, Inaba and non-O1/non-O139 serotypes respectively. The isolates collected in 2014 contained all the three serotypes described in this study. None of the isolates tested belong to the O139 serogroup. Assaying for the presence or absence of 10 known virulence genes, we detected 7 virulence genes among the four 2012 isolates and 9 genes among both the 2014 and 2015 isolates (Table 2). The genes *rtxC* 165/168 (98%) and *tcpP* 50/168 (30%) were the most and least detected respectively. All the 14 non-O1/non-O139 *V*. *cholerae* isolates were negative for the *ctxB* gene. All the 168 isolates harboured at least one virulence gene: however, we did not detect *tagA* in any of the isolates.

**Table 2:** Genotypic analysis of *V*. *cholerae* isolates by year of disease onset.

Gene targeted	2012 (N = 4)	2014 (N = 92)	2015 (N = 72)	Total (N = 168)
	N (%)	N (%)	N (%)	N (%)
***ompW***	4 (100)	92 (100)	72 (100)	168 (100)
***O1-rfb***	4 (100)	85 (92.0)	65 (90.2)	154 (92)
***ctxB***	4 (100)	85 (92.0)	65 (90.2)	154 (92)
***toxR***	4 (100)	84 (91.3)	71 (98.6)	159 (95)
***zot***	4 (100)	77 (83.7)	49 (68.1)	130 (77)
***ace***	4 (100)	72 (78.3)	65 (90.2)	141 (84)
***tcpA***	2 (50.0)	36 (39.1)	62 (86.1)	100 (60)
***rtxA***	4 (100)	74 (80.4)	67 (93.1)	145 (86)
***tcpP***	0 (0.0)	22 (23.9)	28 (38.9)	50 (30)
***hlyA***	4 (100)	81 (88.0)	70 (97.2)	155 (92)
***rtxC***	4 (100)	90 (97.8)	71 (98.6)	165 (98)
***tagA***	0 (0)	0 (0.0)	0 (0.0)	0 (0)

***ompW***-gene encoding outer membrane proteins of *Vibrio cholerae*, ***O1-rfb***- the rfb genes specific for *V*. *cholerae* O1, ***ctx B***-gene encoding the B-subunit of the cholera toxin, ***zot***- gene encoding zonula occluden toxin, ***toxR***- gene that encodes the central regulatory protein toxR, ***ace***- gene encoding accessory cholera enterotoxin, *tcpA*-gene encoding toxin co-regulated pilus A (a colonisation factor), ***tcpP***-gene encoding toxin co-regulated pilus P (a colonisation factor), ***rtxA***- repeat in toxin gene that encode presumptive cytotoxin RTXA, ***rtxC***- a gene that encode RTX cytotoxin activator RTXC, ***hlyA***-gene encoding hemolysin A, ***tagA***-gene that encode TagA lipoprote.

**Fig 3:**
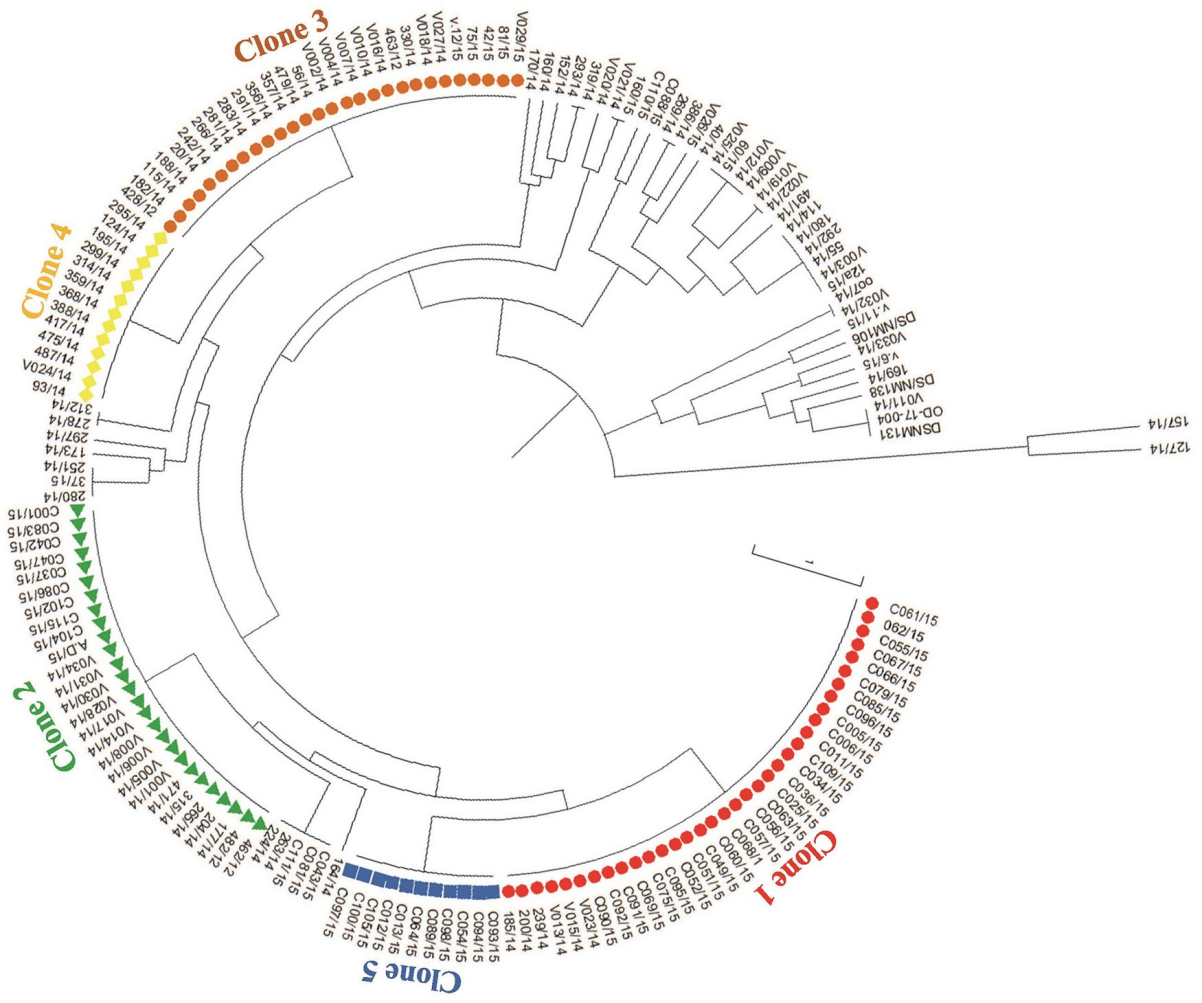
A phylogenetic tree depicting clonal distribution of *V*. *cholerae* isolates based on the distribution of virulence genes. At least five large clones were observed based on the presence or absence of virulence genes. The tree was constructed using the neighbour-joining clustering algorithm.

A cumulative data from the genotypic analysis based on the presence/absence of specific virulence factors was used to determine clonality among the *V*. *cholerae* isolates and depicted five large distinct clones (Fig 3) based on the presence or absence of encoding genes. Of the 10 virulent factors characterized, 6 (*O1*-*rfb*+*ctxB*+*toxR*+*hlyA*+*rtxA*+*rtxC*) were common among all the 5 clones. The highest number of virulence factors was detected in clone 1 (red) (Fig 3, Table 3). None of the 14 non-O1/non-O139 *V*. *cholerae* isolates clustered with any of the 5 large clones, nor clustered together, but constitute singletons.

**Table 3:** Clonal distribution of virulent factors among 2012, 2014 and 2015 outbreak isolates.

CLONE ID	CLONAL PATTERN OF VIRULENT FACTORS
**CLONE 1 (RED)**	*ompW+O1-rfb+ctxB+toxR+hlyA+rtxA+rtxC+zot+ace+tcpA+tcpP*
**CLONE 2 (GREEN)**	*ompW+O1-rfb+ctxB+toxR+hlyA+rtxA+rtxC+zot+ace+tcpA*
**CLONE 3 (BROWN)**	*ompW+O1-rfb+ctxB+toxR+hlyA+rtxA+rtxC+zot+ace*
**CLONE 4 (YELLOW)**	*ompW+O1-rfb+ctxB+toxR+hlyA+rtxA+rtxC+zot+ace +tcpP*
**CLONE 5 (BLUE)**	*ompW+O1-rfb+ctxB+toxR+hlyA+rtxA+rtxC+ace +tcpA+tcpP*

### 3.4. Multi-locus variable number of tandem repeats analysis

**Fig 4:**
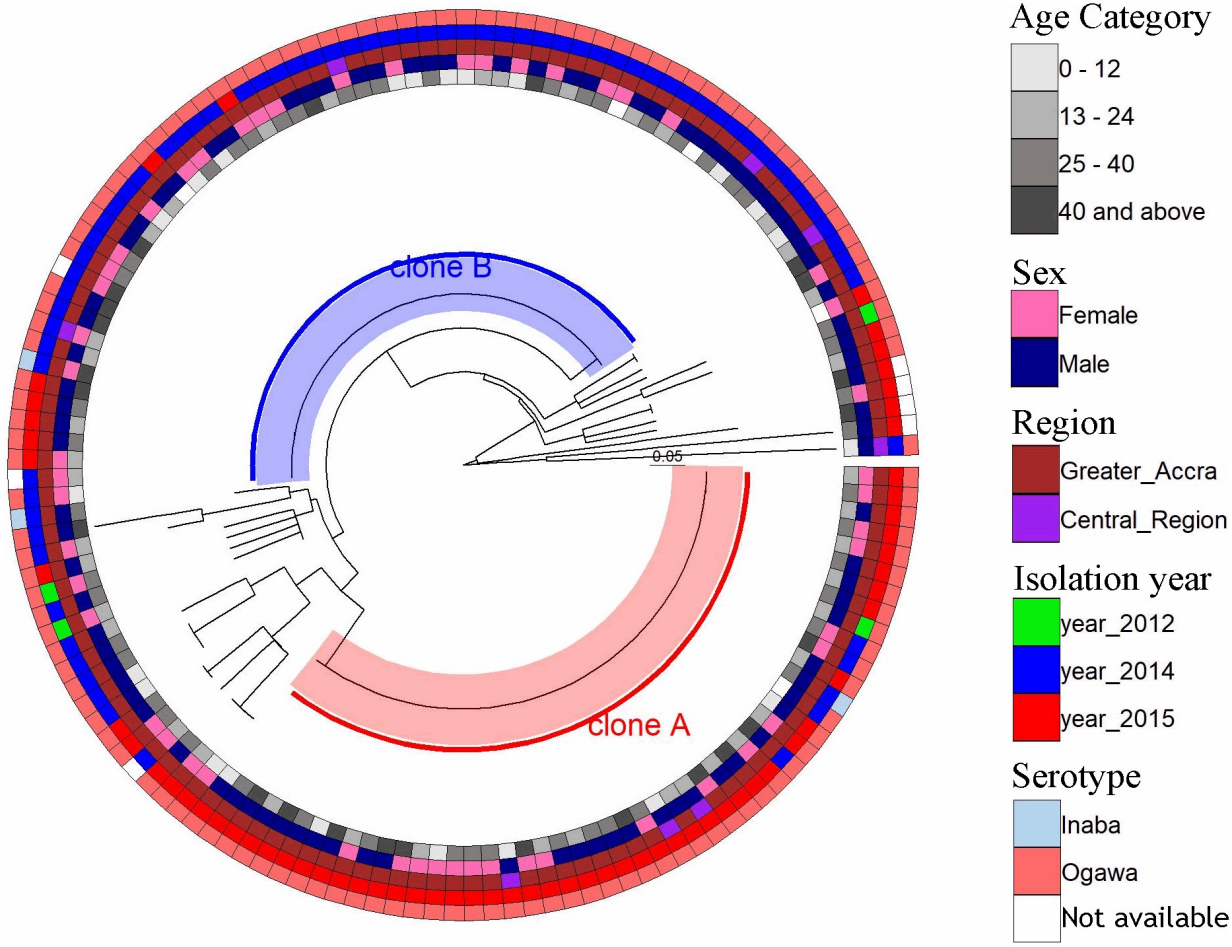
Phylogenetic tree of multi-locus variable number of tandem-repeat (VNTR) analysis (MLVA) for *Vibrio cholerae* isolates by age (inner circle), sex, region isolation year and serotype (outer circle).

**Fig 5:**
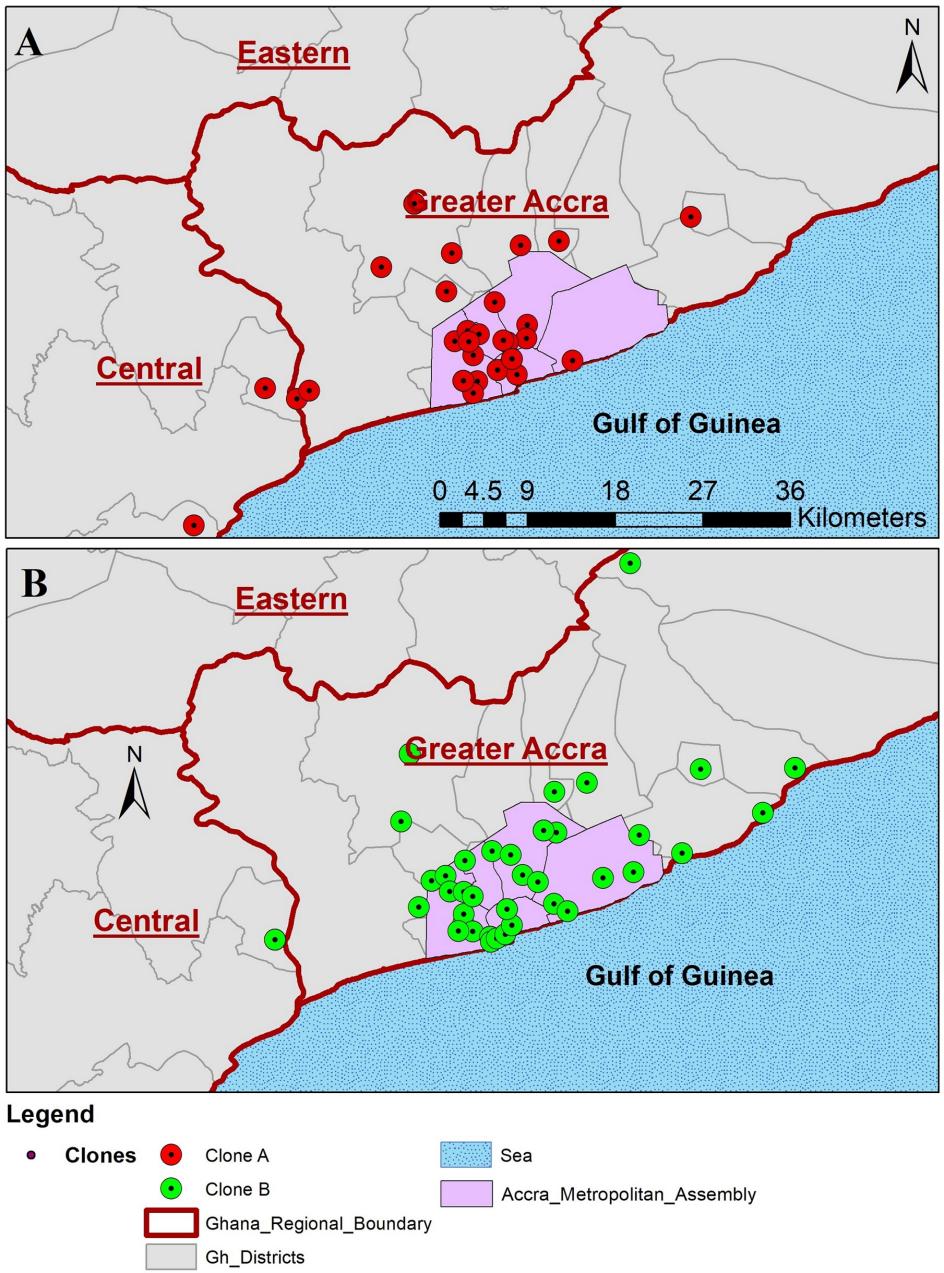
Spatial distribution of *V*. *cholerae* isolates during 2012–2015 outbreak periods. The two large clones obtained from the MLVA data were found to be largely distributed within the heart of Greater Accra region precisely the Accra Metropolitan Assembly (AMA), with a few distributed in the outskirts of AMA. At least four cases from clone A (3 cases) and clone B (1 case) were from the Central region.

Using PCR amplification and DNA sequencing analysis of the five VNTRs (VC0147, VC0436-7, VC1650, VCA0171 and VCA0283), approximately 83.3% (140/168) of the samples analysed gave good sequence data for all the five loci analysed and were used in the MLVA. The most polymorphic VNTRs were VC0171 and VCA0283 with 9 different copy-numbers among the isolates whereas VNTRs VC0436-7 and VC1650 were the least polymorphic with 4 distinct copy-numbers each. Two large unique clusters; cluster A: 50 isolates and cluster B: 59 isolates were observed from the clustering analysis of the combined MLVA data (Fig 4). These clusters were independent of age, sex, facility where samples were collected and virulence marker clonal designations but dependent on the year of isolation. Whereas cluster A was dominated by isolates from the year 2015 except for 5 isolates in 2014 and 1 in 2012, cluster B was dominated by isolates from 2014 with the exception of 6 isolated in 2015. The two large clones obtained from the MLVA data were found to be largely distributed within the heart of Greater Accra region precisely the Accra Metropolitan Assembly (AMA), with a few distributed in the outskirts of AMA (Fig 5). At least four cases from clone A (3 cases) and clone B (1 case) were from the Central region. The two clones were distributed in 15 districts/sub-districts with Ablekuma sub-district of the AMA recording the highest being 22.9% (25/109) followed with Ga West Municipal (16.5%, 18/109).

## 4. Discussion

Our study of archived bacterial isolates from patients with diarrhoea during the 2012, 2014 and 2015 cholera outbreaks in Ghana reveals several important findings. Only 61% of the isolates from the presumed cholera cases were confirmed to be caused by *V*. *cholerae* O1 and pathogens other than vibrios were detected. The frequency and diversity of other pathogens associated with diarrhoea in Ghana, concurs with similar reports from Nigeria, Bangladesh, Tanzania and Sierra Leone [28–31]. This underscores the need to scale up laboratory-based surveillance of diarrhoeal diseases so that accurate identification of the specific aetiology of diarrhoeal diseases can be made using the Integrated Disease Surveillance and Response (IDSR) approach to effectively identify causative agent to enhance appropriate therapy.

Our data shows high resistance patterns to first- and second-line antibiotics recommended by the Ghana Health service for cholera treatment [32], which include nalidixic acid, sulfamethoxazole/trimethoprim and erythromycin, and moderate resistance to doxycycline and chloramphenicol. Similar patterns have been reported from studies in Mozambique and Ghana [33–36], but are in contrast to reports from India and South Mozambique [37, 38]. We also recorded high levels of resistance to erythromycin (69% in 2014 and 58% in 2015) an antibiotic that was previously considered most potent for cholera treatment [39]. In Ghana, antibiotics are easily acquired over-the-counter without prescription and this misuse may be responsible for the continuing emergence of antibiotic resistant strains [40]. Antibiotics are commonly used in animal husbandry as growth supplements to prevent infection and to increase yield. Therefore, residues can readily be passed on to humans when such product is consumed [41, 42]. We detected an increasingly reduced susceptibility to ciprofloxacin (2012, 2014, and 2015), an observation which is contrary to the report by Kuma *et al*. [35].

The pathogenesis of cholera is complex involving several factors that aid *V*. *cholerae* bacteria to colonize and release enterotoxin (CT) that disrupts ion transport by intestinal epithelial cells. Although production of CT, encoded by the *ctxAB* genes, is directly responsible for the manifestation of diarrhoea [43], cholera pathogenesis relies on the synergistic action of additional genes. Our findings suggest that 91.7% of the cases were caused by toxigenic *V*. *cholerae* capable of causing highly dehydrating diarrhoea. The *ctxAB* gene plays a critical role towards the dehydration caused by toxigenic *V*. *cholerae* and is responsible for the pathogenesis. Interestingly, none of the remaining 14 (8.3%) non-O1/O139 *V*. *cholerae* isolates which were also negative for the *ctxB* gene were part of the large clones. Their inability to be part of large clones may be due to the absence of the *ctxB* gene which makes them less virulent. We detected the *tcpA* gene which is a colonization factor in 59% of the isolates suggesting that about 40% of the isolates though may express the cholera toxin might be impaired with their ability to colonize the host. None of our isolates tested positive for serogroup O139 and this finding is concordant with earlier reports of unsuccessful detection of this serogroup from geographical areas outside Asia [44]. We found 14 isolates which tested negative for the serogroup O1 indicating the presence or emergence of *V*. *cholerae* strains other than O1 and O139 causing cholera-like diseases. This finding is consistent with a recent study by Dutta *et al*., which found serogroups other than O1 and O139 causing cholera-like diarrhoea in patients from Kolkata in India [45]. All the isolates tested were predominantly Ogawa serotype, confirming as the main circulating serotype in West Africa [33, 46–48]. We however, found three of our isolates to be Inaba serotype that is mostly found in Asia. It is possible that these resulted from serotype switch from Ogawa to Inaba due to potential protective Ogawa antibodies from previous exposure [49]. Alternatively, the Inaba serotype could have been imported. Thus, this calls for more stringent public health control measures to prevent spread of these virulent strains. Furthermore, our findings support the current WHO licensed cholera vaccines which include both O1 Inaba and Ogawa serotypes.

We found non-toxigenic variants that harboured other virulence genes such as *hlyA*, *rtxC*, *rtxA*, ace and *toxR* but lacked the *ctxB* gene that encodes the cholera toxin in the outbreak isolates. These isolates could be more virulent upon acquisition of cholera toxin in the future, which compares with findings by Eibach *et al*. [34]. MLVA Clustering analysis based on copy-numbers of the 5 VNTRs used for the study identified two major clusters (clusters A and B) of *V*. *cholerae*. These clusters were independent of age, sex or geographical location meaning the only factor that was significantly associated with the clustering was the year of isolation (2014 and 2015). While bacteria of cluster A were mostly responsible for the cholera outbreak in 2015, those of cluster B were responsible for 2014 outbreak cases which suggests an active transmission of *V*. *cholerae* instead of independent cases. Nevertheless, there were few 2014 outbreak genotypes that persisted in 2015 signifying the inefficiency of the control measures put in place or existence of an unknown environmental reservoir. This data agrees with findings of Eibech *et al*. [34] and Abana *et al*. [50], which suggested a potential reservoir in the environment. Interestingly, the clustering pattern by MLVA was very different from the clonal distribution derived from the presence/absence of specific virulent factors (Figs 3 and 4). This highlights the potential confounding due to horizontal gene transfer and the superiority of MLVA for genotyping compared to PCR detection of virulence genes.

There are variable reports on the distribution of cholera outbreaks in space and in time. There is the need to distinguish clonal and multi-clonal outbreaks to guide cholera control measures [51]. Out of the 15 districts within Greater Accra and Central Region (Fig 5), the strains were mostly clustered in the Accra Metropolis mainly within the Ablekuma sub-district. Ablekuma is densely populated and a low socio-economic sub-urban area which may have accounted for the outbreak [52].

We acknowledge that whole genome sequencing (WGS) and analysis of isolates could add more information to the findings. We recognize this as a limitation of the study. Nevertheless, the current study has provided useful information for disease treatment and control, and hope to build on this in a future study to include WGS.

## 5. Conclusions

Cholera outbreaks in Ghana are caused by *Vibrio cholerae* (mostly of the Ogawa serotype). We detected several virulence genes among the *V*. *cholerae* isolates. A high rate of *V*. *cholerae* resistant to first- and second-line antibiotics occurs. The identification of several virulence genes among the two different genotypes of *V*. *cholerae* isolates calls for scaleup of preventive strategies to reduce transmission and strengthening of public health laboratories for rapid antimicrobial susceptibility testing to guide accurate treatment.

## Supporting information

S1 Table Antibiotic susceptibility profile of *Vibrio cholerae* isolates across three outbreak years. (DOCX)
