# Supplementary material for: A molecular and epidemiological study of Vibrio cholerae isolates from cholera outbreaks in southern Ghana
Source: PLoS One. 2020 Jul 10;15(7):e0236016. doi: 10.1371/journal.pone.0236016 (PMC7351161; doi:10.1371/journal.pone.0236016)
Supplement: S1 Table — (DOCX) [file pone.0236016.s001.docx]

**Table S1**: **Antibiotic susceptibility profile of *Vibrio cholerae* isolates across three outbreak years**

| **Antibiotics** | **2012 (N=4)** | | | **2014 (N=92)** | | | **2015 (N=72)** | | |
| --- | --- | --- | --- | --- | --- | --- | --- | --- | --- |
|  | **Sensitive** | **Intermediate** | **Resistant** | **Sensitive** | **Intermediate** | **Resistant** | **Sensitive** | **Intermediate** | **Resistant** |
| **Ampicillin** | 3 (75%) | 1 (25%) | 0 (0%) | 20 (22%) | 10 (11%) | 62 (67%) | 5 (7%) | 15 (21%) | 52 (72%) |
| **Cotrimoxazole** | 0 (0%) | 1 (25%) | 3 (75%) | 7 (8%) | 0 (0%) | 85 (92%) | 5 (7%) | 1 (1%) | 66 (92%) |
| **Gentamicin** | 4 (100%) | 0 (0%) | 0 (0%) | 84 (91%) | 6 (7%) | 2 (2%) | 71 (99%) | 0 (0%) | 1(1%) |
| **Cefuroxime** | 4 (100%) | 0 (0%) | 0 (0%) | 87 (95%) | 5 (5%) | 0 (0%) | 65 (90%) | 4 (6%) | 3 (4%) |
| **Ceftriaxone** | 4 (100%) | 0 (0%) | 0 (0%) | 86 (94%) | 4 (4%) | 2 (2%) | 57 (79%) | 9 (13%) | 6 (8%) |
| **Ciprofloxacin** | 4 (100%) | 0 (0%) | 0 (0%) | 71 (77%) | 2 (2%) | 19 (21%) | 48 (66%) | 12 (17%) | 12 (17%) |
| **Amikacin** | 4 (100%) | 0 (0%) | 0 (0%) | 70 (76%) | 7 (8%) | 15 (16%) | 64 (89%) | 0 (0%) | 8 (11%) |
| **Tetracycline** | 4 (100%) | 0 (0%) | 0 (0%) | 75 (82%) | 5 (5%) | 12 (13%) | 63 (88%) | 1 (1%) | 8 (11%) |
| **Chloramphenicol** | 4 (100%) | 0 (0%) | 0 (0%) | 53 (57%) | 9 (10%) | 30 (33%) | 20 (28%) | 31 (43%) | 21 (29%) |
| **Doxycycline** | 4 (100%) | 0 (0%) | 0 (0%) | 66 (72%) | 1 (1%) | 25 (27%) | 59 (82%) | 3 (4%) | 10 (14%) |
| **Erythromycin** | 4 (100%) | 0 (0%) | 0 (0%) | 5 (5%) | 24 (26%) | 63 (69%) | 23 (32%) | 7 (10%) | 42 (58%) |
| **Nalidixic acid** | 2 (50%) | 1 (25%) | 1 (25%) | 0 (0%) | 4 (4%) | 88 (96%) | 0 (0%) | 5 (7%) | 67 (93%) |
| **Streptomycin** | 3 (75%) | 1 (25%) | 0 (0%) | 3 (3%) | 26 (28%) | 63 (69%) | 6 (8%) | 1 (1%) | 65 (90%) |
| **Flucloxacilin** | 2 (50%) | 2 (50%) | 0 (0%) | 4 (4%) | 21 (23%) | 67 (73%) | 3 (4%) | 1 (1%) | 68 (95%) |
| **Ceftazidime** | 4 (100%) | 0 (0%) | 0 (0%) | 81 (88%) | 8 (9%) | 3 (3%) | 63 (88%) | 1 (1%) | 8 (11%) |
| **Cefotaxime** | 4 (100%) | 0 (0%) | 0 (0%) | 85 (93%) | 4 (4%) | 3 (3%) | 66 (92%) | 2 (2%) | 4 (6%) |
| **Azithromycin** | 4 (100%) | 0 (0%) | 0 (0%) | 90 (98%) | 2 (2%) | 0 (0%) | 61 (85%) | 6 (8%) | 5 (7%) |
